# Dynamic peri-device leak assessment of left atrial appendage occlusion device using super-resolution CT imaging

**DOI:** 10.1007/s10554-026-03653-5

**Published:** 2026-02-20

**Authors:** Prashant Nagpal, Jakub M. Siembida, Martin G. Wagner

**Affiliations:** 1 Department of Radiology, University of Wisconsin School of Medicine and Public Health, Madison, WI USA; 2 Department of Medical Physics, University of Wisconsin, Madison, Wisconsin USA

**Keywords:** Left atrial appendage occlusion, Watchman device, Peri-device leak, Super-resolution CT, Cardiac CT

A 62-year-old male with paroxysmal atrial fibrillation and prior Watchman device implantation was referred for three-month follow-up after device placement. There was no peri-device leak (PDL) reported at the completion of the implantation procedure in the catheterization laboratory. Cardiac CT was performed on a Canon Aquilion ONE / INSIGHT Edition scanner utilizing super-resolution iterative reconstruction (Precise IQ Engine, PIQE) with 1024 matrix. The patient was in sinus rhythm with occasional premature atrial complexes at the time of imaging. The heart rate during acquisition was 78 beats per minute; no heart rate control was administered. The scan was acquired in a single heartbeat using this wide-area detector (320-row) volume scanner, and multiphase reconstruction demonstrated a small PDL that exhibited dynamic behavior across the cardiac cycle (Supplementary Video-1). In diastole (75% R-R) (Fig. 1A), the left atrial (LA) wall was abutting the device, and the PDL location was not apparent. However, in systole (40% R-R) (Fig. 1B), the PDL location became conspicuous due to left atrial wall motion and device-tissue interface changes during cardiac contraction.

**Fig. 1:**
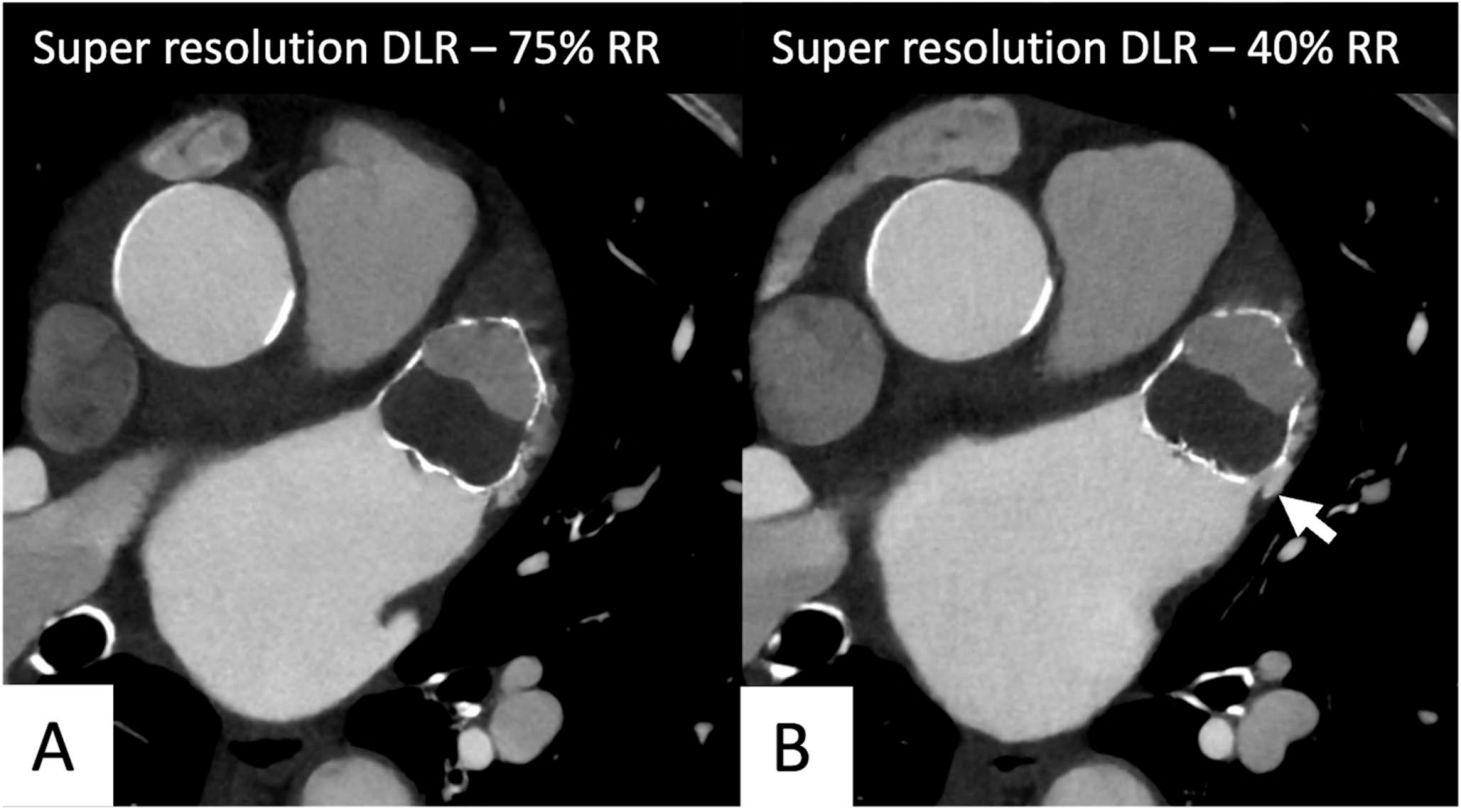
Cardiac CT images demonstrating dynamic peri-device leak following Watchman device implantation. (**A**) Diastolic phase (75% R-R interval) images show the device in appropriate position with presence of contrast in the excluded portion of the left atrial appendage; however, PDL location and size could not be determined. In systolic phase (40% R-R interval, **B**) PDL location (arrows) becomes apparent due to left atrial wall motion

Since the follow-up and management of post-Watchman patients with PDL is based on size of the leak [1], this case illustrates that PDLs demonstrate dynamic variation with the cardiac cycle secondary to atrial wall motion, emphasizing the value of multiphase or wider temporal acquisition windows for accurate detection and sizing. This case also highlights the potential utility of super-resolution or high-resolution imaging techniques with 1024 matrix to enhance visualization of small leaks [2]. The findings from this single case should be interpreted with caution, as the dynamic behavior of PDLs may vary depending on the type and burden of atrial fibrillation, baseline LAA contractile function, cardiac rhythm at the time of imaging, and heart rate. Further investigation in larger patient cohorts is needed to determine whether these observations are generalizable.

## Supplementary Information

Below is the link to the electronic supplementary material.

Supplementary Material 1 Cine loop demonstrating the dynamic nature of the peri-device leak throughout the cardiac cycle, highlighting the temporal variation in PDL conspicuity between systole and diastole.

## Data Availability

No datasets were generated or analysed during the current study.
